# Could Early Surgery Get Beneficial in Adult Patients with Active Native Infective Endocarditis? A Meta-Analysis

**DOI:** 10.1155/2017/3459468

**Published:** 2017-02-23

**Authors:** Liqun Jia, Zanxin Wang, Qiang Fu, Huaien Bu, Minxin Wei

**Affiliations:** ^1^Department of Cardiovascular Surgery, Tianjin Medical University General Hospital, Tianjin 300052, China; ^2^Department of Public Health, Tianjin University of Traditional Chinese Medicine, Tianjin 300193, China

## Abstract

After a thorough search through the database as PubMed database and Embase database, the clinical experimental articles have been selected out on the effects of early surgery on the treatment of active native infective endocarditis. The quality of the trials included in this study was assessed by researcher according to the Cochrane Handbook for Systematic Reviews of Interventions, version 5.1.0. A meta-analysis was carried out in terms of clinical efficacy criteria by RevMan 5.3 software. Based on the results, we cautiously conclude that early surgery used for active native infective endocarditis could reduce in-hospital mortality, follow-up mortality, and IE-related mortality.

## 1. Introduction

The real incidence of infective endocarditis is hard to be obtained because of the population characteristics and other reasons. The incidence would be between 2 and 100 episodes/100,000 person-years [1, 2]. Infective endocarditis (IE) has poor prognosis, with the in-hospital mortality of 20% or even higher [3]. Since the use of penicillin, medical treatment has been performed in IE patients. Although medical treatment reduced the cases of death, the mortality of IE patients was still high. Because of the limitation in medical treatment, surgical therapy has been used for the treatment in some patients with IE. In 1960s, surgical therapy has been performed in IE patients. In the last 3 decades, more and more patients have received surgical treatment. In some western countries, surgery has been performed in around 50% of the patients with IE [4]. Surgery has been the major therapy for IE patients. The main indications for surgery in the treatment of IE were as follows: heart failure from structure destruction, persistent sepsis hardly to be controlled by formal medical therapy, large vegetation with embolism events, cardiac abscesses, ventricular arrhythmia, and so forth. A series of articles has been reported about comparison of outcomes between surgical therapy and medical treatment alone in recent years.

Better outcomes have been reported from surgical therapy in most of the articles. Conventionally, surgery should be performed after 4–6 weeks antibiotic treatment, except in some urgent cases, to reduce recurrence rate. This therapy was defined as “conventional therapy” in some articles. However, there were still many problems. During the long duration of medical treatment before surgery, embolic events, heart failure, formation of abscess cavity, and other complications were observed. And the mortality rate was still high. In consideration of all the problems above, optimal timing for surgery is the focus domain nowadays. In last 3 decades, the idea of “early surgery” has been set up. Although the definition of “early surgery” has not been unified, surgery performed in active phase has been used in some researches as the definition of “early surgery”. Early surgery has been recommended by some articles, with mortality of 6%–25%, and the long-term survival rate was about 70% [5, 6]. It could reduce mortality, embolic events, and improve heart function of patients with IE. And the recurrence rate in early surgery was not elevated. But in some other articles, the results were still debated, with no better prognosis [7]. In consideration of the low incidence of IE and ethics reasons, RCT research in IE patients was rare. Except for one RCT study, the conclusion has been derived mostly from retrospective studies. The conclusion may be influenced by following factors: hidden group, treatment bias, survivor bias, referral bias, and some other confounding factors. Some techniques have been used in reducing bias, such as propensity score analysis, Cox proportional hazards regression technique, and Biprobit technique. However, these methods were all statistical techniques and could not resolve all the drawbacks above. As a result, it was still controversial in whether early surgical therapy could result in better outcomes. And in 2015 ESC guidelines for the management of IE, although early surgery was recommended, the level of evidence was still level B [8].

For the above considerations, meta-analysis could make efforts in this domain. There has been some meta-analysis articles published years before, and no articles with high quality were published recently. In the concern of different mortality between native valve endocarditis (NVE) and prosthetic valve endocarditis (PVE), we focused on the effect of surgical and early surgical therapy for the patients with NVE. Meta-analysis has been used in our study to estimate the effect of early surgical therapy for the patients with NVE.

## 2. Methods

### 2.1. Search Strategy

PubMed and Embase databases were searched for English and Chinese language including the articles published during January 1990 to June 2015. The following Medical Subject Heading terms and/or keywords were used as “endocarditis”, “early surgery”, and “infective endocarditis surgery”. Studies comparing early surgery with conventional therapy in patients with IE were included. Two authors reviewed the trials, ensured that they met inclusion criteria, and abstracted the data.

### 2.2. Eligibility Criteria

#### 2.2.1. Types of Studies

We included randomized controlled trials and retrospective study.

#### 2.2.2. Participants

The patients diagnosed with active native infective endocarditis clearly.

#### 2.2.3. Interventions

Early surgery was defined as follows: (1) surgery performed in active phase; (2) the duration between surgery and entrance to hospital was not more than 4 weeks; and (3) surgery performed during initial hospitalization.

#### 2.2.4. Outcomes Measures

The outcomes assessed in this meta-analysis are the mortality rates of IE patients in different groups.

### 2.3. Data Extraction and Quality Assessment

The qualities of the data were assessed by two independent researchers. The third researcher would be invited for discussion whenever different opinions appeared. The quality of the trials included in this study was assessed by each researcher according to the Cochrane Handbook for Systematic Reviews of Interventions, version 5.1.0.

### 2.4. Statistical Analysis

Risk ratio (RR) and 95% confidence intervals (CI) were used for counting data as effect size. Chi-square test would be applied to the heterogeneity. Fixed-effect model would be adopted when *P* > 0.1 or *I*^2^ < 50%. Random-effect model would be used when *P* < 0.1 or *I*^2^ > 50%. The statistics analysis was performed with Review Manager 5.3.

## 3. Results

### 3.1. Description of the Included Trails

#### 3.1.1. Study Selection

**Figure 1:**
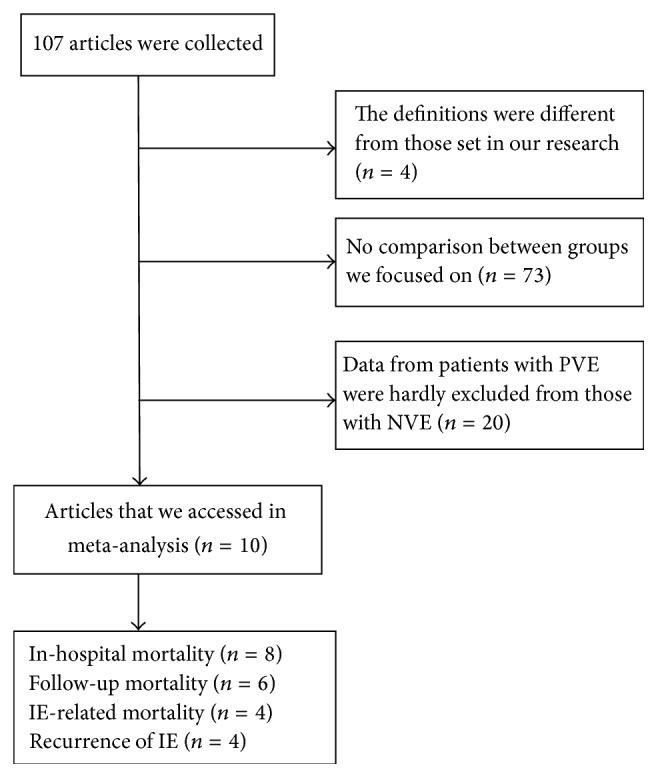
Flow chart of literature retrieval and trial selection.

A total of 107 reports were identified by our electronic database search and through other sources. After removing articles with different definitions (*n* = 4), without comparison between groups (*n* = 73), with the data from PVE patients that could not be excluded from NVE patients (*n* = 20). Finally, ten studies [9–18] met our inclusion criteria and were included in the present analysis (Figure 1).

#### 3.1.2. Characteristics of Included Studies

They are shown in Table 1.

#### 3.1.3. Quality of the Included Studies

It is shown in Table 2.

### 3.2. The Effect of Early Surgery

#### 3.2.1. In-Hospital Mortality

**Figure 2:**
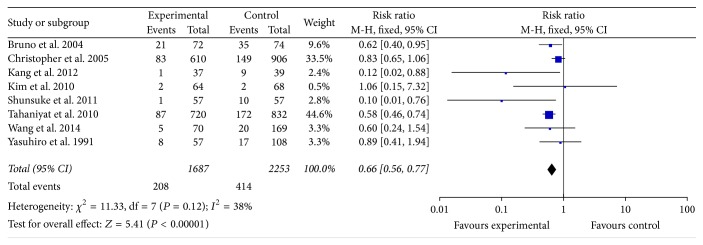
In-hospital mortality between two groups.

The in-hospital mortality was reported in 8 studies that involved 3940 participants. All of these studies reported in-hospital mortality with early surgery compared with conventional medicine. Some of these studies reported evidence that early surgery reduced in-hospital mortality (RR = 0.66, 95% CI; 0.56, 0.77). There was no heterogeneity among the 8 studies (*P* = 0.12, *I*^2^ = 38%) (Figure 2).

#### 3.2.2. Follow-Up Mortality

**Figure 3:**
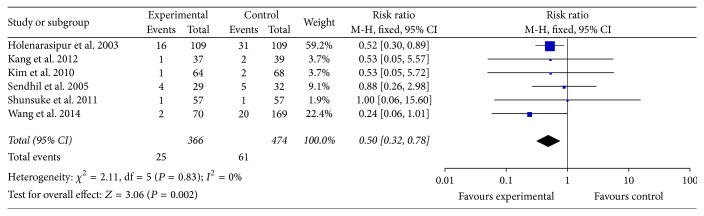
Follow-up mortality between two groups.

The follow-up mortality was reported in 6 studies that involved 840 participants. All of these studies reported follow-up mortality with early surgery compared with conventional medicine. Some of these studies reported evidence that early surgery reduced follow-up mortality (RR = 0.50, 95% CI; 0.32, 0.78). There was no heterogeneity among the 6 studies (*P* = 0.83, *I*^2^ = 0%) (Figure 3).

#### 3.2.3. IE-Related Mortality

**Figure 4:**
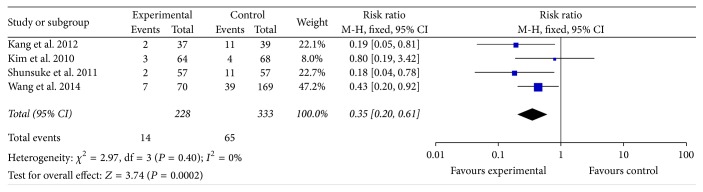
IE-related mortality between two groups.

The IE-related mortality was reported in 4 studies that involved 561 participants. All of these studies reported IE-related mortality with early surgery compared with conventional medicine. Some of these studies reported evidence that early surgery reduced IE-related mortality (RR = 0.35, 95% CI; 0.20, 0.61). There was no heterogeneity among the 6 studies (*P* = 0.40, *I*^2^ = 0%) (Figure 4).

#### 3.2.4. Recurrence of IE

**Figure 5:**
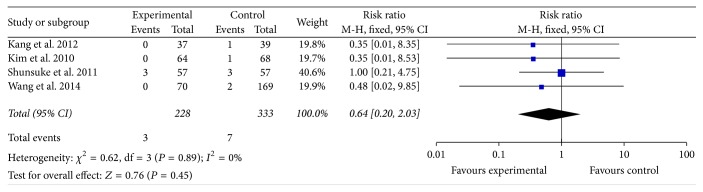
Recurrence of IE between two groups.

The recurrence of IE was reported in 4 studies that involved 561 participants. All of these studies reported recurrence of IE with early surgery compared with conventional medicine. The result showed that early surgery was no better or worse at reducing recurrence of IE (RR = 0.64, 95% CI; 0.20, 2.03). There was no heterogeneity among the 4 studies (*P* = 0.89, *I*^2^ = 0%) (Figure 5).

### 3.3. Funnel Plot of Publication Bias

**Figure 6:**
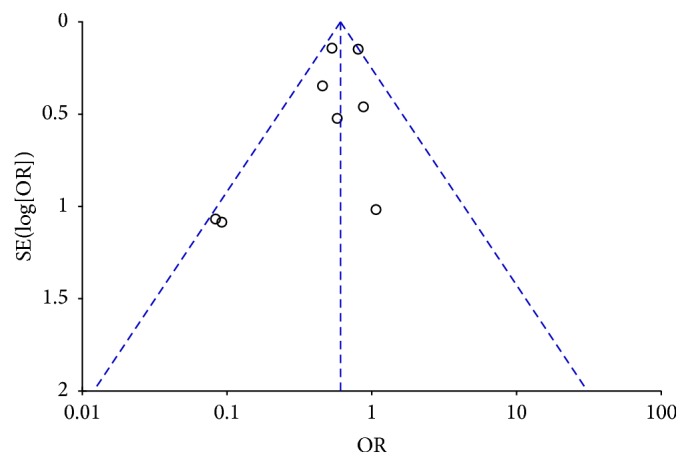
Funnel plot.

Funnel plot analysis was conducted based on eight studies included, and this plot is summarized in Figure 6. The outcome suggests that there was little publication bias.

## 4. Discussion

### 4.1. Efficacy Analysis of Early Surgery

In Figure 2, the in-hospital mortality in early surgery group was as 66% as that in conventional group. In Figure 3, the follow-up mortality in early surgery group was only as half as that in conventional group. We could also find that the IE-related mortality in early surgery group was as 35% as that in conventional group from Figure 4. From all the mentioned above, patients in early surgery group had better outcome than those in conventional group. In propensity matched cohort study, the mortality of patients in early surgery group also seems to be lower.

These results are consistent with some other studies published before [19–21]. As mentioned in the articles, the baseline characteristics are different between the two groups. In early surgery group, more patients get severe cardiac damage, heart failure, and abscesses. And in conventional therapy group, patients get more coexisting diseases, like cerebral vascular disease, diabetes mellitus, and so forth. This is consistent with the report from Mourvillier et al.

Heart failure could be found in 42–60% of IE patients [22], and nearly 60% of IE patients received surgical treatment because of heart failure from structure destruction [23]. As reported before, patients with IE, with indication for surgical treatment, would get better cardiac function by early surgery [24]. In conventional therapy group, patients who should get surgical treatment usually get surgery after 4–6 weeks' medical treatment. During this period, structure destruction would develop, like aortic valve and mitral valve regurgitation. Destruction of cardiac structure would lead to deterioration of cardiac function. Patients with cardiac abscesses could also get benefit from early surgery. Furthermore, many cardiac abscesses and fistulae are found during surgery, without any presurgery evidence. And early surgery could get much benefit in these patients, in avoiding of the fatal condition. As a result, early surgery could get better cardiac function. Patients in early surgery group would get lower mortality rate for this reason.

Furthermore, embolic events, with the incidence of even up to 50%, are severe complications of IE, including cerebral embolism, kidney embolism, and spleen embolism. Embolic events are mostly found two weeks after discovery of cardiac vegetations [25, 26]. After the antibiotic duration of 4–6 weeks, the incidence of embolic events would get high, especially in the patients with silent embolism. Because of these conditions, early surgery could reduce the incidence of embolic events, which is consistent with Kang et al.'s report [12], and could lead to the lower mortality rate.

Nowadays, diagnostic methods and medical treatments in IE patients have developed much. No promotion of recurrence rate of IE had been observed in early surgery group in Figure 5. These results denoted that early surgery in NVE patients would be safe, and long-term antibiotic treatment would get no benefit.

### 4.2. Limitation

The included studies were mainly retrospective study; confounding factors may limit our interpretation. Considerable differences in baseline characteristics exist among the included studies, including regional variations and microbiological differences, and heterogeneity in clinical trials also existed. In our results, statistical heterogeneity was relatively small, with the *I*^2^ as follows: 38% in in-hospital mortality, 0% in follow-up mortality, 0% in IE-related mortality, and 0% in recurrence of IE. As in the situations mentioned above, there was heterogeneity in our research; however, most researches denoted early surgery therapy would get better outcome. As a result, early surgery would reduce mortality rate in IE patients. Among the articles that we have read, which focus on this controversial subject, our present study would be the first meta-analysis which makes comparison of the outcomes between early surgery therapy and conventional therapy in the patients with native valve infective endocarditis. Our research suggests the superiority of early surgery therapy. This finding should be used as hypothesis generating and as basis for further well designed randomized trials.

### 4.3. Prospects for Early Surgery

As mentioned above, early surgery could get better cardiac function, lower embolic events, and lower mortality. Recurrence of IE is catastrophic. Nowadays, antimicrobial therapy has developed greatly, and the methods of investigation of pathogens have been much developed. PCR technique has also been used to find pathogens [27]. Early surgery will not elevate the incidence of reinfection, which has been proved by some researches [12]. As a result, long-term duration is not necessary in respect of reinfection.

According to our results, early surgery in NVE patients will not elevate the incidence of reinfection and reduce mortality rate. This finding is consistent with the recommendations in 2015 ESC IE guidelines and should be used as basis for further well designed researches.

## 5. Conclusion

The current limited evidence showed that when compared with conventional medicine, early surgery could reduce in-hospital mortality, follow-up mortality, and IE-related mortality.

## Figures and Tables

**Table 1:** Characteristics of the 10 trials.

Studies	Sample (T/C)	Diagnosis standard	Age (T/C)	Intervention group	Control group	Follow-up (median, year)	Outcome
Holenarasipur et al. 2003	109/109	Modified Duke criteria	53 ± 15/55 ± 19	Early surgery	Conventional treatment	0.5	Follow-up mortality
Kim et al. 2010	64/68	Modified Duke criteria	45.9 ± 15.9/51.1 ± 17.4	Early surgery	Conventional treatment	3.8	In-hospital mortality; follow-up mortality; IE-related mortality; recurrence of IE
Wang et al. 2014	70/169	Modified Duke criteria	41.6 ± 12.0/45.6 ± 17.2	Early surgery	Conventional treatment	2	In-hospital mortality; follow-up mortality; IE-related mortality; recurrence of IE
Kang et al. 2012	37/39	Modified Duke criteria	45.5 ± 14.9/47.8 ± 17.5	Early surgery	Conventional treatment	0.5	In-hospital mortality; follow-up mortality; IE-related mortality; recurrence of IE
Shunsuke et al. 2011	57/57	Modified Duke criteria	55 ± 18/53 ± 17	Early surgery	Conventional treatment	5.5	In-hospital mortality; follow-up mortality; IE-related mortality; recurrence of IE
Tahaniyat et al. 2010	720/832	Modified Duke criteria	53/61	Early surgery	Conventional treatment	Unclear	In-hospital mortality
Christopher et al. 2005	610/906	Modified Duke criteria	54.7 ± 15.2/61.1 ± 17.4	Early surgery	Conventional treatment	Unclear	In-hospital mortality
Sendhil et al. 2005	29/32	Modified Duke criteria	22–80/23–80	Received antibiotics for <2 weeks before surgery	Received antibiotics for 2–4 weeks before surgery	3.1	Follow-up mortality
Bruno et al. 2004	72/74	Modified Duke criteria	Unclear	Early surgery	Conventional treatment	Unclear	In-hospital mortality
Yasuhiro et al. 1991	57/108	O'Brien Pesanti	15.9 (0,85)	Early surgery	Conventional treatment	Unclear	In-hospital mortality

**Table 2:** Quality of the included studies.

Studies	Random sequence generation	Allocation concealment	Blinding	Incomplete outcome data	Selective reporting	Other bias
Holenarasipur et al. 2003	High risk	Unclear	Unclear	Low risk	Low risk	Low risk
Kim et al. 2010	High risk	High risk	Unclear	Low risk	Low risk	Low risk
Wang et al. 2014	High risk	Unclear	Unclear	Low risk	Low risk	Low risk
Kang et al. 2012	Low risk	Unclear	Unclear	Low risk	Low risk	Low risk
Shunsuke et al. 2011	High risk	Unclear	Unclear	Low risk	Low risk	Low risk
Tahaniyat et al. 2010	High risk	Unclear	Unclear	Low risk	Low risk	Low risk
Christopher et al. 2005	Unclear	Unclear	Unclear	Low risk	Low risk	Low risk
Sendhil et al. 2005	Unclear	Unclear	Unclear	Low risk	Low risk	Low risk
Bruno et al. 2004	Unclear	Unclear	Unclear	Low risk	Low risk	Low risk
Yasuhiro et al. 1991	Unclear	Unclear	Unclear	Low risk	Low risk	Low risk
